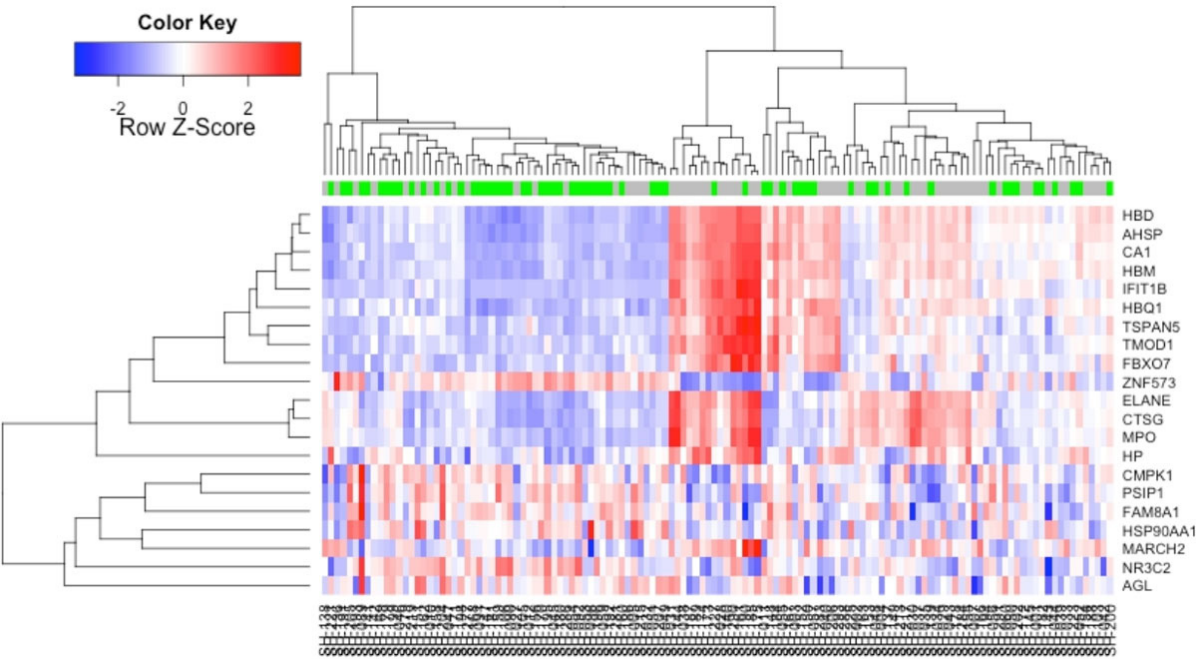# Association of haptoglobin genetic polymorphism with overall survival in advanced castration-resistant prostate cancer patients with personalized peptide vaccination

**DOI:** 10.1186/2051-1426-1-S1-P56

**Published:** 2013-11-07

**Authors:** Nobukazu Komatsu, Xiaoliang Pang, Hiromitsu Araki, Yoshiro Koda, Kimiko Soejima, Tetsuro Sasada, Kosuke Tashiro, Satoru Kuhara, Kyogo Itoh

**Affiliations:** 1 Kurume University, Kurume-shi, Japan; 2 Kyushu University, Fukuoka-shi, Japan

## 

Although a peptide vaccination is potentially useful for cancers, there are some patients who do not show clinically beneficial response to this treatment. Previously, we have demonstrated gene expression profiling of pre-vaccination peripheral blood mononuclear cells in advanced castration-resistant prostate cancer patients with personalized peptide vaccination, and we have found some genes related with overall survival. In this study, we sequenced the upstream region of Haptoglobin (HP), which is the most significant q-value in a univariate Cox regression analysis (q-value=0.001) and highly expressed in patients with poor clinical response. Genetic polymorphism of the upstream of HP was identified and this polymorphism classifies patients with good or poor clinical response to a peptide vaccination. Our results suggested that mRNA level and genetic polymorphism of HP is related to the prognosis of advanced castration-resistant prostate cancer patients treated with peptide vaccination.

**Figure 1:**